# Adjustment of Surveillance Intervals for Ulcerative Colitis‐Associated Neoplasia Based on Disease Duration

**DOI:** 10.1111/den.15073

**Published:** 2025-06-19

**Authors:** Ryoya Sakakibara, Shinya Sugimoto, Yuta Kaieda, Hiroki Kiyohara, Yusuke Yoshimatsu, Kaoru Takabayashi, Soichiro Murakami, Miho Kawaida, Tomohisa Sujino, Naoki Hosoe, Motohiko Kato, Yasushi Iwao, Yohei Mikami, Takanori Kanai

**Affiliations:** ^1^ Division of Gastroenterology and Hepatology, Department of Internal Medicine Keio University School of Medicine Tokyo Japan; ^2^ Center for Diagnostic and Therapeutic Endoscopy Keio University School of Medicine Tokyo Japan; ^3^ Department of Pathology Keio University School of Medicine Tokyo Japan; ^4^ Center for Preventive Medicine Keio University Tokyo Japan

**Keywords:** colitis‐associated neoplasms, colorectal cancer, dysplasia, surveillance

## Abstract

**Objectives:**

The risk of colitis‐associated cancer increases with disease duration in ulcerative colitis (UC), yet surveillance colonoscopy protocols generally stratify risk uniformly for patients with disease lasting over 8 years. This study evaluated whether shorter surveillance intervals might enhance lesion detection rates in patients with extended disease duration.

**Methods:**

This retrospective observational study analyzed patients diagnosed with UC‐associated neoplasms between 2010 and 2023. Colonoscopies before lesion detection were retrospectively reviewed, and risk stratification was applied according to four established guidelines. The recommended surveillance intervals were recalculated based on the stratified risk, and lesion detection rates were compared across increasing risk categories for specific disease duration.

**Results:**

A total of 39 patients were included, with a median disease duration of 21 years (14–27), and a median colonoscopy interval of 1.3 years (1.1–2.2). Lesion detection rates were 72%, 59%, 44%, and 56% for American Society for Gastrointestinal Endoscopy (ASGE), American Gastroenterological Association (AGA), European Crohn's and Colitis Organisation (ECCO), and British Society of Gastroenterology (BSG) guidelines, respectively. Adjusting risk stratification upward by one rank for disease durations of ≥ 15, ≥ 20, ≥ 25, and ≥ 30 years resulted in increased detection rates: 90%, 87%, 82%, and 72% for ASGE; 85%, 82%, 74%, and 64% for AGA; 82%, 74%, 62%, and 49% for ECCO; and 82%, 79%, 72%, and 62% for BSG, respectively. The longest period with a significant difference in detection rates was observed in patients with disease durations of 20–25 years.

**Conclusions:**

For patients with long‐standing UC, reducing surveillance intervals may improve the detection of colitis‐associated neoplasia, with a practical focus on those with 20–25 years of disease duration.

**Abbreviations**
AGA: American Gastroenterological Association
ASGE: American Society for Gastrointestinal Endoscopy
BSG: British Society of Gastroenterology
*D*_det_
: date of UCAN detection
*D*_pre_
: date of previous colonoscopy
*D*_rec_
: date of recommendation for next colonoscopy
ECCO: European Crohn's and Colitis Organisation
UC: ulcerative colitis
UCAN: ulcerative colitis‐associated neoplasia

## 1 Introduction

Ulcerative colitis (UC) is a chronic idiopathic inflammatory bowel disease that affects the colorectum and is associated with an increased risk of colitis‐associated cancers, a severe complication arising from chronic inflammation that significantly impacts patient outcomes [1]. UC‐associated neoplasia (UCAN) is characterized by a distinct p53‐mediated carcinogenic pathway that differs from that of sporadic colorectal cancers. UCAN lesions are frequently flat and poorly defined, complicating early detection efforts [2]. Histologically, these lesions often exhibit invasive features associated with poor prognoses, with outcomes worsening significantly when diagnosed at advanced stages [3]. The critical importance of early detection and timely therapeutic intervention for UCAN has underscored the value of surveillance colonoscopy in patients with UC. Current clinical guidelines recommend initiating surveillance programs for patients with a disease duration of at least 8 years [4, 5, 6, 7, 8, 9]. However, the optimal interval for surveillance colonoscopy remains undefined, with existing guidelines offering recommendations that are not strongly supported by high‐quality evidence. Chronic inflammation in UC has been shown to promote genetic mutations and carcinogenesis [10], with the risk increasing proportionally to the disease duration [11]. Despite this, surveillance intervals are stratified based on known risk factors for carcinogenesis, while the uniform criterion for initiating surveillance—disease duration of 8 years or more—may not adequately account for individual patient risk. Current surveillance guidelines fail to consider disease duration as a dynamic, individualized risk factor, creating gaps in knowledge about the practical implementation and effectiveness of surveillance colonoscopy programs. In this study, we aimed to evaluate the impact of existing surveillance practices on the detection of UCAN lesions using data from our institutional UCAN cohort. Furthermore, we investigated the potential role of disease duration as an independent risk factor in improving the effectiveness of surveillance for UCAN.

## 2 Methods

### 2.1 Patient Selection and Data Collection

This study was conducted as a retrospective observational investigation that included patients diagnosed with UC‐associated high‐grade dysplasia or adenocarcinoma at Keio University Hospital, Tokyo, Japan, between September 2010 and February 2023. As documented in prior studies [2, 3, 12, 13, 14, 15, 16], the included cases were diagnosed endoscopically and confirmed as UCAN by final histopathological evaluation performed by at least two experienced pathologists with expertise in UCAN. Patients with the following diagnoses without UCAN were excluded from the cohort: non‐dysplastic lesions, neuroendocrine tumors, sporadic adenomas/carcinomas, serrated lesions, traditional serrated adenomas, and indefinite UCAN. Comprehensive demographic and clinical data were collected, including patient sex, age at UCAN detection and at one prior colonoscopy, duration of UC, disease extent, anatomical location of UCAN, and the presence of concomitant primary sclerosing cholangitis. Exclusion criteria were strictly applied to ensure cohort uniformity. Patients with a UC duration of < 8 years at the time of colonoscopy prior to UCAN diagnosis were excluded. Similarly, patients lacking complete colonoscopic imaging from the preceding examination in which UCAN was identified were not included. Furthermore, patients undergoing follow‐up at intervals of < 1 year—either for disease monitoring or after the detection of low‐grade dysplasia that eventually progressed to high‐grade dysplasia or adenocarcinoma—were excluded, as such cases did not align with the study's objectives, which focused on standard surveillance practices.

### 2.2 Evaluation of Endoscopic and Histologic Findings

The diagnostic approach for UCAN, integrating histological assessment with advanced endoscopic techniques, such as indigo carmine dye spraying and magnifying endoscopy, has been previously reported in the literature [2, 16]. In the present study, UCAN cohort data diagnosed by these established methods were used. Detailed procedures are presented in Data S1.

### 2.3 Assumption of Lesion Identification Rate

**FIGURE 1:**
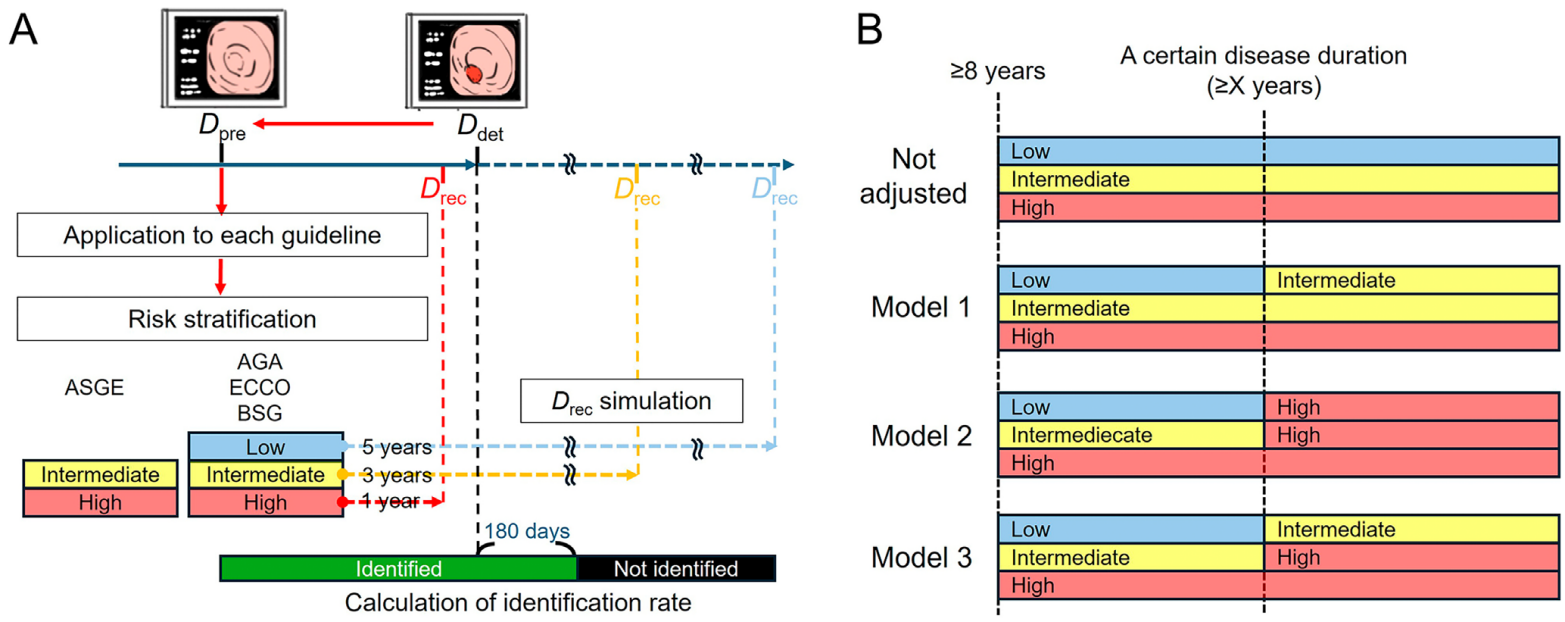
Schematics of lesion identification rate assumptions. (A) Assumptions of lesion identification rates based on each guideline. (B) Risk‐adjusted assumptions using three models based on disease duration. The risk level increases by one if the disease duration exceeds *X* years. AGA, American Gastroenterological Association; ASGE, American Society for Gastrointestinal Endoscopy; BSG, British Society of Gastroenterology; *D*_pre_, date of previous colonoscopy; *D*_det_, date of UCAN detection; *D*_rec_, date of recommendation for next colonoscopy; ECCO, European Crohn's and Colitis Organisation.

The analysis included cases with available data from both the most recent colonoscopy in which UCAN was not diagnosed and the first colonoscopy in which UCAN was detected. The date of the former examination was designated as the date of the previous colonoscopy (*D*_pre_), whereas the latter was defined as the date of UCAN detection (*D*_det_). To evaluate whether lesions could be identified within the surveillance intervals recommended by various guidelines, the recommended date for the next colonoscopy (*D*_rec_) was calculated on the basis of *D*_pre_ (Figure 1A). Patient risk stratification for UCAN was conducted according to the American Society for Gastrointestinal Endoscopy (ASGE) [4] guidelines, which classified risk into two levels, and the American Gastroenterological Association (AGA) [5], European Crohn's and Colitis Organisation (ECCO) [6], and British Society of Gastroenterology (BSG) [7] guidelines, each of which stratified risk into three levels (Table 1). The Japanese Society for Gastrointestinal Endoscopy [8] and the American College of Gastroenterology [9] guidelines, which do not specify intervals based on stratified risk levels, were excluded from this analysis. Across the included guidelines, common high‐risk criteria included severe disease activity, complications of primary sclerosing cholangitis, bowel stenosis, and a family history of early‐onset colorectal cancer. For high‐risk cases meeting any of these criteria, *D*_rec_ was uniformly set as 1 year after *D*_pre_. For guidelines specifying a surveillance interval range (e.g., 1–3 years or 2–3 years), *D*_rec_ was standardized to 3 years after *D*_pre_ to distinguish from the 1‐year surveillance interval of the high‐risk group. For guidelines with three risk levels, *D*_rec_ for the low‐risk group, defined by the absence of risk factors, was set at 5 years after *D*_pre_. If *D*_rec_ fell within 180 days of *D*_det_, the endoscopic interval was classified as successful for lesion identification. In cases where *D*_rec_ preceded *D*_det_ by 1 year, lesion identification was also deemed successful, as a subsequent *D*_rec_ would occur in the following year. On the basis of these criteria, lesion identification rates were calculated for each guideline, accounting for their respective risk stratification frameworks and surveillance interval recommendations.

**TABLE 1:** Summary of recommendations in each guideline for endoscopy surveillance intervals.

	ASGE	AGA	ECCO	BSG
Recommended timing of starting surveillance colonoscopy (after diagnosis; years)	8	8–10	8	8
Low risk				
Recommended interval (years)	Not defined	5	5	5
Risk factor				
No active inflammation^a^	✓	✓	✓	✓
Intermediate risk				
Recommended interval (years)	1–3^b^	2–3^b^	2–3^b^	3
Risk factors				
Mild inflammation		✓	✓	✓
Moderate inflammation			✓	
Family history of colorectal cancer in first degree relative age > 50 years		✓	✓	✓
Moderate pseudopolyposis		✓		✓^c^
Mucosal scarring		✓		
History of lower risk visible dysplasia < 5 years ago		✓		
High risk				
Recommended interval (years)	1	1	1	1
Risk factors				
Mild inflammation	✓			
Moderate inflammation	✓	✓	✓	✓
Severe inflammation	✓	✓	✓	✓
Family history of colorectal cancer in first degree relative age < 50	✓^d^	✓	✓	✓
Primary sclerosing cholangitis	✓	✓	✓	✓
Stricture	✓		✓^e^	✓^e^
History of invisible dysplasia or higher‐risk visible dysplasia < 5 years ago	✓	✓	✓	✓
Dense pseudopolyposis	✓	✓		

^a^ AGA: Continuous disease remission since last colonoscopy with mucosal healing on the current examination, plus either of the following: ≥ 2 consecutive examinations without dysplasia, minimal historical colitis extent (ulcerative proctitis). ECCO: Extensive colitis with minimal endoscopic or histological inflammation, Colitis affecting < 50% of the colon. BSG: No active endoscopic or histological inflammation, left‐sided colitis.

^b^ In this study, defined as 3 years.

^c^ Regardless of degree.

^d^ Regardless of age at diagnosis.

^e^ In the past 5 years.

### 2.4 Risk‐Adjusted Assumptions Based on Disease Duration

Three models were evaluated to assess alterations in lesion identification rates following adjustments for disease duration as a risk factor (Figure 1B). The duration of disease was categorized into four threshold patterns: ≥ 15, ≥ 20, ≥ 25, and ≥ 30 years. In Model 1, cases exceeding a specific disease duration were classified as intermediate risk, with a reassessment interval of 3 years. In Model 2, cases were classified as high risk, necessitating annual surveillance, a strategy deemed neither cost‐effective nor practical. To address this limitation, Model 3 introduced a stepwise risk escalation, increasing the risk level by one tier (e.g., from low risk to intermediate risk or from intermediate risk to high risk). Each model incorporated risk adjustments based on disease duration, and corresponding changes in the *D*_rec_ were calculated. These calculations also accounted for the adjusted lesion identification rates under each model.

### 2.5 Statistical Analysis

Continuous variables were reported as medians with interquartile ranges. Categorical variables were summarized as percentages. Intergroup comparisons were made using Pearson's chi‐squared test or Fisher's exact test. Comparisons between the original lesion identification rates for UCAN and the adjusted rates under each model were performed using the McNemar test, which was also applied to determine the statistical significance of differences in lesion identification rates across varying disease durations. Two‐sided *p* values < 0.05 were considered statistically significant.

## 3 Results

### 3.1 Patient Profile

**FIGURE 2:**
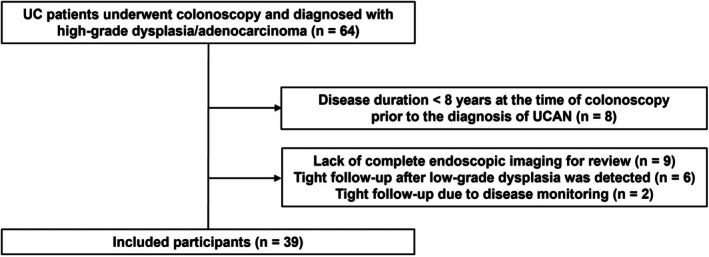
Patient flow through the study. Thirty‐nine of 64 patients with ulcerative colitis and high‐grade dysplasia/adenocarcinoma were enrolled.

A total of 64 patients who underwent colonoscopy at our institution between September 2010 and February 2023 were diagnosed with UC‐associated high‐grade dysplasia or adenocarcinoma. Among these, exclusions were applied to patients with a duration of UC < 8 years at *D*_pre_ (*n* = 8; 12.5%), those with a short interval between colonoscopies (*n* = 8), and those for whom complete endoscopic imaging data at *D*_pre_ were unavailable for review (*n* = 9). After these exclusions, a total of 39 patients were included in the analysis (Figure 2).

### 3.2 Patient Characteristics

**FIGURE 3:**
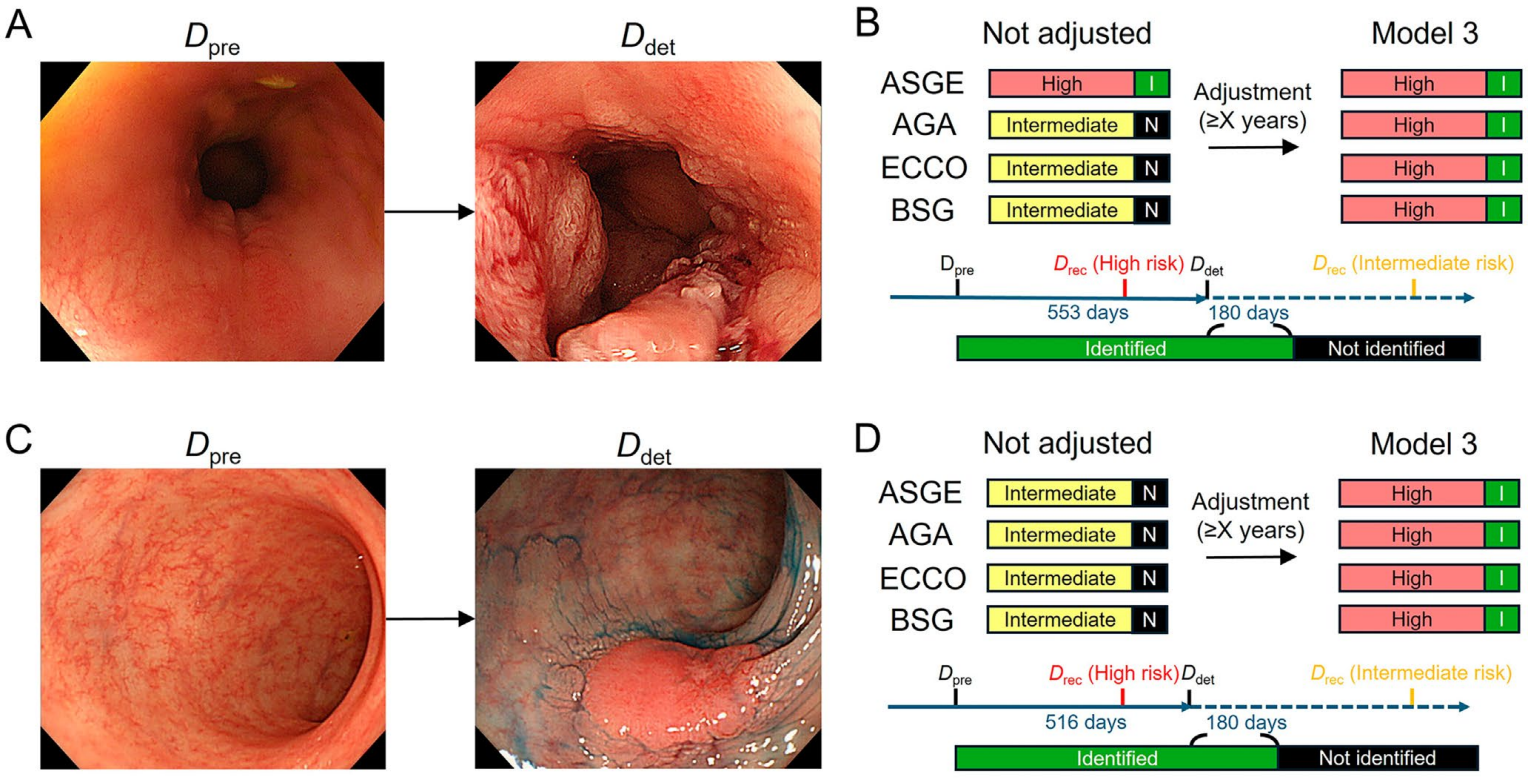
Representative endoscopic images before and after UCAN detection. (A, C) Endoscopic images at the time of lesion detection (*D*_det_, right) and one prior to detection (*D*_pre_, left). (A, B) Disease duration at *D*_det_: 40 years, corresponding to an intermediate‐risk case. Examination interval: 19 months. The lesion, located in the rectum, was diagnosed as moderately differentiated adenocarcinoma with a T3 depth. (C, D) Disease duration at *D*_det_: 27 years, corresponding to an intermediate‐risk case. Examination interval: 17 months. The lesion, located in the sigmoid colon, was diagnosed as well‐differentiated adenocarcinoma with a T1 depth. (B, D) Schematics of the time course and risk stratification. *D*_rec_ values are shown for the original and Model 3, adjusted for disease durations exceeding *X*+ years. *X* varies depending on the adjustment: for *X* = 15, 20, 25, or 30, B shifts to high risk; for *X* = 15, 20, or 25, D shifts to high risk. Assumptions for lesions identified (I, green) and not identified (N, black) are displayed. AGA, American Gastroenterological Association; ASGE, American Society for Gastrointestinal Endoscopy; BSG, British Society of Gastroenterology; *D*_det_, date of UCAN detection; *D*_pre_, date of previous colonoscopy; *D*_rec_, date of recommendation for next colonoscopy; ECCO, European Crohn's and Colitis Organisation; I, identified; N, not identified.

The demographic and clinical characteristics of the 39 patients with UCAN are summarized in Tables 2, 3 and S1. The cohort consisted of 27 men (69%) and 12 women (31%), with a median age at UCAN diagnosis of 52 years (46–67 years). The median duration of UC was 22 years (16–30 years), with a median disease duration of 21 years (14–27 years) at *D*_pre_. The median colonoscopy interval (*D*_det_ − *D*_pre_) was 1.3 years (1.1–2.2 years). When stratified by lesion depth, no significant differences in colonoscopy interval were observed: Tis lesions (*n* = 19) had a median interval of 1.3 years (1.1–2.2 years); T1 lesions (*n* = 6), 1.5 years (1.2–1.8 years); T2 lesions (*n* = 4), 1.2 years (1.0–1.7 years); and lesions deeper than T3 (*n* = 10), 1.3 years (1.0–1.5 years). Of the included patients, six (15%) did not meet the surveillance interval criteria recommended by established guidelines. Among the 33 surveillance colonoscopies conducted within recommended intervals, 16 (48%) detected lesions with a depth greater than T1 (Figure 3). Eleven patients (28%) were in both endoscopic and histologic remission at the time of assessment.

**TABLE 2:** Characteristics of 39 lesions with high‐grade dysplasia/adenocarcinoma.

Morphology	
SCENIC classification	
Pedunculated, *n* (%)	1 (3)
Sessile, *n* (%)	3 (8)
Superficial elevated, *n* (%)	10 (26)
Flat, *n* (%)	6 (15)
Depressed, *n* (%)	10 (26)
Macroscopic type	
1 (Polypoid type), *n* (%)	1 (3)
2 (Ulcerative type with clear margin), *n* (%)	1 (3)
3 (Ulcerative type with infiltration), *n* (%)	2 (5)
4 (Diffusely infiltrating type), *n* (%)	0 (0)
5 (Unclassified type), *n* (%)	5 (13)
Tumor size, mm, median (IQR)	28 (17–40)
Location	
Cecum, *n* (%)	0 (0)
Ascending, *n* (%)	1 (3)
Transverse, *n* (%)	2 (5)
Descending, *n* (%)	2 (5)
Sigmoid, *n* (%)	15 (38)
Rectum, *n* (%)	19 (49)
Depth	
Intramucosa (M, Tis), *n* (%)	19 (49)
Submucosa (SM, T1), *n* (%)	6 (15)
Muscularis propria (MP, T2), *n* (%)	4 (10)
Subserosa or Adventitia (SS or A, T3), *n* (%)	5 (13)
Serosa (SE, T4) *n* (%)	5 (13)
Histologic type	
High‐grade dysplasia,^a^ *n* (%)	18 (46)
tub, *n* (%)	13 (33)
por/sig/muc, *n* (%)	8 (21)
p53 immunostaining	
Positive, *n* (%)	32 (82)
Complete absence, *n* (%)	7 (18)

^a^ Although neoplasms with nuclear and architectural abnormalities are diagnosed as intramucosal carcinoma regardless of invasion status in Japan, these lesions were classified as high‐grade dysplasia in this study.

**TABLE 3:** Characteristics of 39 patients with ulcerative colitis and high‐grade dysplasia/adenocarcinoma.

Male/Female, *n* (%)/*n* (%)	27 (69)/12 (31)
Age, years, median (IQR)	52 (46–67)
Disease duration, years, median (IQR)	22 (16–30)
8–14, *n* (%)	10 (26)
15–19, *n* (%)	9 (23)
20–24, *n* (%)	7 (18)
25–29, *n* (%)	6 (15)
30 or more, *n* (%)	7 (18)
Surveillance interval, years, median (IQR)	1.3 (1.1–2.2)
Mayo endoscopic subscore	
0, *n* (%)	20 (51)
1, *n* (%)	12 (31)
2, *n* (%)	5 (13)
3, *n* (%)	2 (5)
Concomitant primary sclerosing cholangitis, *n* (%)	0 (0)
Family history of colorectal cancer, *n* (%)	0 (0)
Stricture, *n* (%)	2 (5)
Pseudopolyposis, *n* (%)	10 (26)
Mucosal scarring, *n* (%)	20 (51)
Dysplasia < 5 years, *n* (%)	4 (10)
Extent	
Pancolitis, *n* (%)	31 (79)
Left‐sided colitis, *n* (%)	8 (21)
Proctitis, *n* (%)	0 (0)
Medical history of ulcerative colitis	
Mesalamine, *n* (%)	37 (95)
Thiopurines, *n* (%)	17 (44)
Prednisolone, *n* (%)	1 (3)
Infliximab, *n* (%)	2 (5)
Adalimumab, *n* (%)	1 (3)
Vedolizumab, *n*, (%)	2 (5)
Prior use of prednisolone, *n* (%)	17 (44)
Prior use of infliximab, *n* (%)	2 (5)
Prior use of tacrolimus, *n* (%)	3 (8)
Prior use of cytapheresis, *n* (%)	6 (15)

Abbreviation: IQR, interquartile range.

### 3.3 Adjustment of Surveillance Intervals by Disease Duration Increases Lesion Identification Rate

**FIGURE 4:**
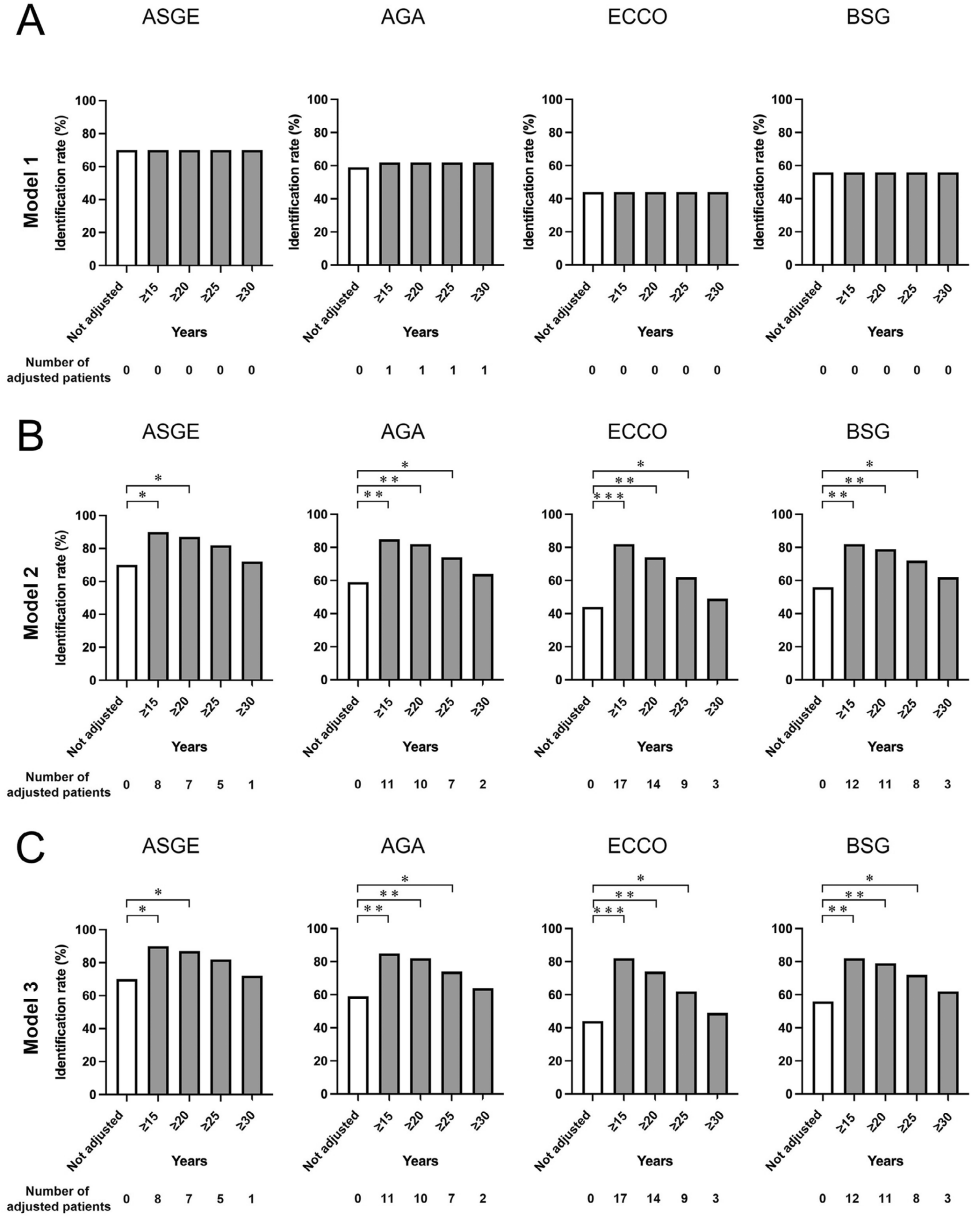
Lesion identification rates by surveillance interval assumptions across guidelines. Comparison of lesion identification rates before and after adjustments using Model 1 (A), Model 2 (B), and Model 3 (C). McNemar test: *p < 0.05; **p < 0.01; ***p < 0.001. AGA, American Gastroenterological Association; ASGE, American Society for Gastrointestinal Endoscopy; BSG, British Society of Gastroenterology; ECCO, European Crohn's and Colitis Organisation.

Risk stratification based on the criteria outlined in each guideline demonstrated that the intermediate‐ and high‐risk groups constituted the majority of cases (Table 4). A comparison of the *D*_rec_ determined by guideline‐specific risk stratification, starting from *D*_pre_ to the actual *D*_det_, revealed lesion identification rates of 72% for ASGE, 59% for AGA, 44% for ECCO, and 56% for BSG guidelines. These were used as baseline data and adjusted for the duration of disease (Figure 4).

**TABLE 4:** Number of patients in each risk group before and after adjustment according to each guideline.

	Not adjusted				Model 1			Model 2			Model 3		
	Low	Intermediate	High	Adjustment	Low	Intermediate	High	Low	Intermediate	High	Low	Intermediate	High
ASGE	n/a	10	29	≥ 15 years	n/a	10	29	n/a	2	37	n/a	2	37
				≥ 20 years		10	29		3	36		3	36
				≥ 25 years		10	29		5	34		5	34
				≥ 30 years		10	29		9	30		9	30
AGA	2	13	24	≥ 15 years	1	14	24	1	3	35	1	4	34
				≥ 20 years	1	14	24	1	4	34	1	5	33
				≥ 25 years	1	14	24	1	7	31	1	8	30
				≥ 30 years	1	14	24	1	12	26	1	13	25
ECCO	0	23	16	≥ 15 years	0	23	16	0	6	33	0	6	33
				≥ 20 years	0	23	16	0	9	30	0	9	30
				≥ 25 years	0	23	16	0	14	25	0	14	25
				≥ 30 years	0	23	16	0	20	19	0	20	19
BSG	0	17	22	≥ 15 years	0	17	22	0	5	34	0	5	34
				≥ 20 years	0	17	22	0	6	33	0	6	33
				≥ 25 years	0	17	22	0	9	30	0	9	30
				≥ 30 years	0	17	22	0	14	25	0	14	25

Abbreviations: AGA, American Gastroenterological Association; ASGE, American Society for Gastrointestinal Endoscopy; BSG, British Society of Gastroenterology; ECCO, European Crohn's and Colitis Organisation; n/a, not applicable.

Model 1 was initially applied, categorizing patients with disease durations of ≥ 15 years (*n* = 29), ≥ 20 (*n* = 20), ≥ 25 (*n* = 13), and ≥ 30 (*n* = 7) as intermediate risk. However, given the paucity of low‐risk cases in the cohort, no significant changes in lesion identification rates were observed, precluding meaningful analysis (Table 4, Figure 4A). Subsequently, Model 2 was implemented, reclassifying patients with disease durations of ≥ 15, ≥ 20, ≥ 25, or ≥ 30 years as high risk. For instance, under the ASGE guideline, the lesion identification rate was initially 72% (28/39). Reclassification of eight patients with disease durations ≥ 15 years (7/29 identified lesions) led to an adjusted identification rate of 90% (35/39). Corresponding rates for durations of ≥ 20, ≥ 25, and ≥ 30 years were 87%, 82%, and 72%, respectively. Similarly, risk reassessments under other guidelines yielded increased identification rates, as follows: 90%, 87%, 82%, and 72% for ASGE; 85%, 82%, 74%, and 64% for AGA; 82%, 74%, 62%, and 49% for ECCO; and 82%, 79%, 72%, and 62% for BSG (Figures 1 and 4B, Table 4). These findings indicate that shortened surveillance intervals in patients with prolonged disease durations were associated with significantly improved lesion identification rates.

Using Model 3, which increased the risk category by one rank for disease durations of ≥ 15, ≥ 20, ≥ 25, or ≥ 30 years (Figures 1 and 3), lesion identification rates mirrored those of Model 2. Specifically, rates for ASGE were 90%, 87%, 82%, and 72%; for AGA, 85%, 82%, 74%, and 64%; for ECCO, 82%, 74%, 62%, and 49%; and for BSG, 82%, 79%, 72%, and 62%, respectively (Figure 4C). Because low‐risk participants were almost absent in this cohort, the results for Models 2 and 3 were largely identical. Compared with pre‐adjustment rates, significant differences in lesion identification were noted for ASGE (≥ 15 years: *p* = 0.023; ≥ 20 years: *p* = 0.041), AGA (≥ 15 years: *p* = 0.004; ≥ 20 years: *p* = 0.008; ≥ 25 years: *p* = 0.041), ECCO (≥ 15 years: *p* < 0.001; ≥ 20 years: *p* = 0.002; ≥ 25 years: *p* = 0.023), and BSG (≥ 15 years: *p* = 0.004; ≥ 20 years: *p* = 0.008; ≥ 25 years: *p* = 0.041). These results indicate that in our cohort, increasing risk stratification levels and shortening surveillance intervals for patients with disease durations of ≥ 20–25 years significantly enhanced lesion identification rates, regardless of the guideline applied.

## 4 Discussion

This study evaluated the role of guideline‐directed surveillance endoscopy interval in patients with UCAN and demonstrated that incorporating disease duration as a risk factor for reassessment may enhance lesion detection. Prolonged disease duration is a well‐established risk factor for carcinogenesis in UC [11], with advanced cases and highly invasive histological features frequently observed at the time of lesion detection [3]. In this study, the median interval for surveillance colonoscopy was 1.3 years, and the colonoscopy intervals were consistent across various depths of advanced lesions. These findings align with prior reports [17, 18], suggesting that approximately half of the UCAN detected through surveillance exhibited T1 invasion or deeper, underscoring the challenges of achieving early diagnosis through surveillance colonoscopy. In a nationwide Japanese survey, 27% of surveillance‐detected UCANs were Stage II–IV [18], whereas in our cohort, 10 cases (26%) were Stage II–IV, yielding comparable results. These findings highlight the need to tailor surveillance intervals to mitigate the risk of missed lesions, including interval cancers. Despite variability across existing guidelines, only 44%–72% of lesions in our cohort were identified on the basis of conventional risk stratification criteria. To improve lesion identification rates, we examined the role of disease duration, currently considered only when it exceeds 8 years, without further stratification based on length [19]. Given the established association between disease duration and carcinogenesis, we hypothesized that incorporating disease duration as a stratification factor could optimize risk assessment.

Regarding enhanced surveillance intervals, we anticipated that shorter intervals would improve lesion detection rates. However, risk stratification must balance the benefits of early detection against the burden and cost‐effectiveness of more frequent examinations. Using adjusted models based on current guidelines, we explored three approaches to incorporating disease duration into risk assessment. In Model 1, most patients in the cohort already had intermediate‐ or high‐risk factors, with no significant changes in risk stratification. In Model 2, prolonged disease duration was treated as a high‐risk factor, leading to a significant increase in lesion identification rates. However, this approach was deemed impractical because of the frequency of annual surveillance endoscopies, which deviates from the principles of risk‐based stratification. In Model 3, we refined the approach by elevating patients' risk classification by one level after a specified duration threshold. This strategy effectively reduced the target population for enhanced surveillance while improving lesion detection rates. Importantly, patients initially classified as low‐risk under conventional guidelines were unlikely to develop UCAN, and this study did not assess the impact of reclassifying low‐risk patients as intermediate risk. This finding reinforces the adequacy of current guidelines in distinguishing low‐risk individuals from higher‐risk groups. The most prolonged period with a significant difference in lesion detection rates, adjusted for long disease duration, was observed for patients with durations of 20–25 years. In addition to the small sample size of patients with durations of more than 30 years, the majority had already fulfilled high‐risk criteria and were categorized as high‐risk prior to adjustment. Consequently, many remained in the same risk category following adjustment (Table S2). This threshold appears to be clinically reasonable and could serve as a practical basis for future risk stratification strategies. By reclassifying intermediate‐risk patients as high risk after a prolonged disease duration, surveillance efforts can be more effectively focused, thereby optimizing resource utilization and improving patient outcomes.

This study had several limitations. First, the analysis was based on retrospectively collected descriptive data from a small cohort of patients at a single center. Despite this limitation, to our knowledge, no prior studies have explored clinical data using the specific inspection intervals employed here, as determined by individual guidelines. Second, the absence of a control group without UCAN underscores the need for further studies with larger, more comprehensive datasets that include all patients subject to surveillance. Such data would allow for more robust validation of the findings presented here. Furthermore, the enhanced surveillance strategies proposed could potentially increase the population requiring surveillance colonoscopy. The applicability of our conclusions to elderly patients, particularly those over 80, has not been validated. Balancing an optimal detection rate with a manageable surveillance population size remains a critical issue for future investigation. Third, the study did not standardize endoscopic equipment, examination techniques, or biopsy protocols, introducing variability into the findings. Additionally, the dataset includes a significant number of older cases, and the application of chromoendoscopy—now recommended for surveillance [20]—was limited, except in more recent cases. Nonetheless, this limitation reflects real‐world clinical practice, where the adoption of chromoendoscopy remains inconsistent, even in recent years [21]. Depending on the technical level of the endoscopist and recent advances in treatment, it may not be possible to directly generalize these results. Finally, this study focused exclusively on the detection of high‐grade UCAN and did not address surveillance for low‐grade dysplasia or sporadic lesions in patients with UC. Selection bias may exist because only high‐risk UCANs were selected and due to variation among pathologists in distinguishing UCAN from sporadic neoplasia.

In conclusion, our findings suggest that surveillance colonoscopy, particularly when accounting for disease duration as a key risk factor, may enhance the detection of UCAN. However, even with routine surveillance protocols, early detection of UCAN remains suboptimal. These findings emphasize the importance of endoscopists recognizing the need for intensified surveillance in patients with UC, particularly those with a disease duration exceeding 20–25 years. Prospective studies are warranted to further validate these findings and refine surveillance protocols.

## Author Contributions

**Ryoya Sakakibara:** data curation (lead), formal analysis (lead), investigation (lead), methodology (equal), visualization (equal), writing – original draft preparation (equal). **Shinya Sugimoto:** conceptualization (lead), data curation (equal), investigation (equal), methodology (lead), project administration (lead), visualization (lead), writing – original draft preparation (lead), writing – review and editing (lead). Yuta Kaieda: investigation (supporting). **Yusuke Yoshimatsu:** investigation (supporting). **Soichiro Murakami:** investigation (supporting). **Tomohisa Sujino:** investigation (supporting). **Naoki Hosoe:** investigation (supporting). **Hiroki Kiyohara:** investigation (supporting), methodology (supporting), writing – review and editing (supporting). **Kaoru Takabayashi:** investigation (supporting), writing – review and editing (supporting). **Miho Kawaida:** investigation (supporting), writing – review and editing (supporting). **Motohiko Kato:** resources (supporting), writing – review and editing (supporting). **Yasushi Iwao:** investigation (supporting), resources (equal). **Yohei Mikami:** investigation (supporting), visualization (supporting), writing – review and editing (supporting). **Takanori Kanai:** resources (lead), supervision (lead).

## Ethics Statement

Approval of the research protocol by an Institutional Review Board: The study protocol was reviewed and approved by the Ethics Committee of Keio University School of Medicine (approval number: 20150100).

Informed consent: Witten informed consent was waived.

Registry and the Registration No. of the Study/Trial: None.

Animal Studies: None.

## Conflicts of Interest

The authors declare no conflicts of interest.

## Supporting information


**Data S1** Details of evaluation of endoscopic findings and histologic diagnoses.

**Table S1** Comparative characteristics of the 39 lesions included in the study, stratified by early‐stage neoplasia and advanced cancer.
**Table S2** Assumed lesion identification rates in each guideline based on disease duration.

## References

[1] O. Olén , R. Erichsen , M. C. Sachs , et al., “Colorectal Cancer in Ulcerative Colitis: A Scandinavian Population‐Based Cohort Study,” Lancet 395 (2020): 123–131. 31929014 10.1016/S0140-6736(19)32545-0

[2] S. Sugimoto , M. Naganuma , Y. Iwao , et al., “Endoscopic Morphologic Features of Ulcerative Colitis‐Associated Dysplasia Classified According to the Scenic Consensus Statement,” Gastrointestinal Endoscopy 85 (2017): 639–646.e2. 27884517 10.1016/j.gie.2016.11.013

[3] R. Sakakibara , S. Sugimoto , K. Takabayashi , et al., “Ulcerative Colitis‐Associated Neoplasms Often Harbor Poor Prognostic Histologic Components With Low Detection by Biopsy,” Intestinal Research 22 (2024): 428–438. 38712359 10.5217/ir.2024.00006 PMC11534447

[4] American Society for Gastrointestinal Endoscopy Standards of Practice C , A. K. Shergill , J. R. Lightdale , et al., “The Role of Endoscopy in Inflammatory Bowel Disease,” Gastrointestinal Endoscopy 81 (2015): 1101–1121. e1–13. 25800660 10.1016/j.gie.2014.10.030

[5] S. K. Murthy , J. D. Feuerstein , G. C. Nguyen , and F. S. Velayos , “AGA Clinical Practice Update on Endoscopic Surveillance and Management of Colorectal Dysplasia in Inflammatory Bowel Diseases: Expert Review,” Gastroenterology 161 (2021): 1043–1051.e4. 34416977 10.1053/j.gastro.2021.05.063

[6] H. Gordon , L. Biancone , G. Fiorino , et al., “ECCO Guidelines on Inflammatory Bowel Disease and Malignancies,” Journal of Crohn's and Colitis 17 (2023): 827–854. 10.1093/ecco-jcc/jjac187 36528797

[7] C. A. Lamb , N. A. Kennedy , T. Raine , et al., “British Society of Gastroenterology Consensus Guidelines on the Management of Inflammatory Bowel Disease in Adults,” Gut 68 (2019): s1–s106. 31562236 10.1136/gutjnl-2019-318484 PMC6872448

[8] H. Nakase , M. Uchino , S. Shinzaki , et al., “Evidence‐Based Clinical Practice Guidelines for Inflammatory Bowel Disease 2020,” Journal of Gastroenterology 56 (2021): 489–526. 33885977 10.1007/s00535-021-01784-1 PMC8137635

[9] D. T. Rubin , A. N. Ananthakrishnan , C. A. Siegel , B. G. Sauer , and M. D. Long , “ACG Clinical Guideline: Ulcerative Colitis in Adults,” American Journal of Gastroenterology 114 (2019): 384–413. 30840605 10.14309/ajg.0000000000000152

[10] K. Nanki , M. Fujii , M. Shimokawa , et al., “Somatic Inflammatory Gene Mutations in Human Ulcerative Colitis Epithelium,” Nature 577 (2020): 254–259. 31853059 10.1038/s41586-019-1844-5

[11] J. A. Eaden , K. R. Abrams , and J. F. Mayberry , “The Risk of Colorectal Cancer in Ulcerative Colitis: A meta‐Analysis,” Gut 48 (2001): 526–535. 11247898 10.1136/gut.48.4.526 PMC1728259

[12] S. Sugimoto , M. Shimoda , Y. Iwao , et al., “Intramucosal Poorly Differentiated and Signet‐Ring Cell Components in Patients With Ulcerative Colitis‐Associated High‐Grade Dysplasia,” Digestive Endoscopy 31 (2019): 706–711. 31278777 10.1111/den.13482

[13] M. Mutaguchi , M. Naganuma , S. Sugimoto , et al., “Difference in the Clinical Characteristic and Prognosis of Colitis‐Associated Cancer and Sporadic Neoplasia in Ulcerative Colitis Patients,” Digestive and Liver Disease 51 (2019): 1257–1264. 31151895 10.1016/j.dld.2019.05.003

[14] A. Ikebata , M. Shimoda , K. Okabayashi , et al., “Demarcated Redness Associated With Increased Vascular Density/Size: A Useful Marker of Flat‐Type Dysplasia in Patients With Ulcerative Colitis,” Endoscopy International Open 9 (2021): E552–E561. 33860072 10.1055/a-1352-2709 PMC8041573

[15] S. Sugimoto , Y. Iwao , M. Shimoda , et al., “Epithelium Replacement Contributes to Field Expansion of Squamous Epithelium and Ulcerative Colitis‐Associated Neoplasia,” Gastroenterology 162 (2022): 334–337.e5. 34597671 10.1053/j.gastro.2021.09.051

[16] K. Takabayashi , S. Sugimoto , K. Nanki , et al., “Characteristics of Flat‐Type Ulcerative Colitis‐Associated Neoplasia on Chromoendoscopic Imaging With Indigo Carmine Dye Spraying,” Digestive Endoscopy 36 (2024): 446–454. 37389858 10.1111/den.14628 PMC12136265

[17] K. Hata , H. Anzai , H. Ikeuchi , et al., “Surveillance Colonoscopy for Ulcerative Colitis‐Associated Colorectal Cancer Offers Better Overall Survival in Real‐World Surgically Resected Cases,” American Journal of Gastroenterology 114 (2019): 483–489. 30747769 10.14309/ajg.0000000000000117

[18] T. Noguchi , S. Ishihara , M. Uchino , et al., “Clinical Features and Oncological Outcomes of Intestinal Cancers Associated With Ulcerative Colitis and Crohn's Disease,” Journal of Gastroenterology 58 (2023): 14–24. 36182971 10.1007/s00535-022-01927-y

[19] S. C. Shah and S. H. Itzkowitz , “Colorectal Cancer in Inflammatory Bowel Disease: Mechanisms and Management,” Gastroenterology 162 (2022): 715–730.e3. 34757143 10.1053/j.gastro.2021.10.035 PMC9003896

[20] L. Laine , T. Kaltenbach , A. Barkun , K. R. McQuaid , V. Subramanian , and R. Soetikno , “SCENIC International Consensus Statement on Surveillance and Management of Dysplasia in Inflammatory Bowel Disease,” Gastroenterology 148 (2015): 639–651.e28. 25702852 10.1053/j.gastro.2015.01.031

[21] Z. R. Gallinger , A. Rumman , S. K. Murthy , and G. C. Nguyen , “Perspectives on Endoscopic Surveillance of Dysplasia in Inflammatory Bowel Disease: A Survey of Academic Gastroenterologists,” Endoscopy International Open 5 (2017): E974–E979. 28983504 10.1055/s-0043-117944 PMC5628049

