# Supplementary material for: Adjustment of Surveillance Intervals for Ulcerative Colitis‐Associated Neoplasia Based on Disease Duration
Source: Dig Endosc. 2025 Jun 19;37(10):1068–77. doi: 10.1111/den.15073 (PMC12511916; doi:10.1111/den.15073)
Supplement: Supplementary file 1 — Data S1 Details of evaluation of endoscopic findings and histologic diagnoses. [file DEN-37-1068-s001.docx]

**Supplementary document:**

***Details of evaluation of endoscopic findings***

Surveillance colonoscopy was routinely recommended at 1–2 year intervals for patients with a history of UC exceeding 8 years, provided there were no contraindications, such as procedural risks or patient refusal. At our institution, neoplastic lesions during routine surveillance were identified via targeted biopsies performed using high-definition video colonoscopes (PCF-H290ZI, CF-HQ290I, CF-H260AZI, PCF-H290I, CF-H290I, PCF-Q260AI, CF-H260AI [Olympus, Tokyo, Japan], or EC-L600ZP7 [Fujifilm, Tokyo, Japan]). Suspected lesion sites were further assessed using 0.1%–0.2% indigo carmine dye spraying, magnifying endoscopy, and image-enhanced endoscopy (narrow band imaging [Olympus], texture and color enhancement imaging [Olympus] and/or blue laser imaging [Fujifilm] depending on each endoscopist and equipment), following protocols outlined in previous studies. ^1, 2^ Ten endoscopists were involved in the detection and diagnosis of UCAN. All of them were Board Certified Fellows of the Japan Gastroenterological Endoscopy Society (JGES), and nine have since become Board Certified IBD Trainers of the Japanese Society for Inflammatory Bowel Disease (JSIBD). Additionally, 13 endoscopists were involved in the most recent colonoscopy prior to diagnosis (D_pre_), of whom 12 were Board Certified Fellows of the JGES, and seven are now certified as IBD Trainers by the JSIBD. Endoscopic findings were retrospectively analyzed in accordance with the SCENIC consensus statement for endoscopic morphology, ^3^ the Mayo endoscopic subscoring system for disease activity, and the presence of additional features such as strictures, pseudopolyposis, and mucosal scarring. ^4-7^ Endoscopic remission was defined as the Mayo endoscopic subscore of 0 or 1. Evaluations were finalized through consensus among multiple experienced endoscopists, each with expertise in evaluating over 2,000 UC-related colonoscopy cases.

***Details of histologic diagnoses***

The definitive diagnosis of UCAN was established through histologic evaluation of the entirety of endoscopically or surgically resected lesions by at least two experienced pathologists with expertise in UCAN. An exception was made for three patients who did not undergo resection because of disseminated advanced cancer; in these cases, biopsy specimens and ascites fluid were analyzed for histological assessment. The diagnosis was confirmed via routine hematoxylin-eosin staining, complemented by immunohistochemical analysis of p53 expression patterns (either diffuse or unique basal overexpression, cytoplasmic localization, or complete absence), as well as the presence of UC-associated inflammation and dysplasia in the tissue surrounding the lesion. Ki67 expression patterns, either diffuse or showing a distinct “bottom-up” growth pattern, were also considered when necessary to differentiate sporadic neoplasia, in accordance with prior descriptions. ^1, 8-10^ Lesions in which Ki67 was predominantly expressed at the luminal aspect of the elevated areas, with low levels of p53, were classified as sporadic neoplasia. Cases of sporadic colorectal cancer or adenoma, as well as indefinite UCAN where sporadic lesions could not be ruled out, were excluded. Additionally, biopsy specimens from prior examinations were evaluated using the Geboes scoring system. ^11^ Histologic remission was defined as a Geboes score of <3. ^12^

**Supplementary references**

1 Sugimoto S, Naganuma M, Iwao Y *et al.* Endoscopic morphologic features of ulcerative colitis-associated dysplasia classified according to the scenic consensus statement. *Gastrointest Endosc* 2017; **85**: 639-46 e2.

2 Takabayashi K, Sugimoto S, Nanki K *et al.* Characteristics of flat-type ulcerative colitis-associated neoplasia on chromoendoscopic imaging with indigo carmine dye spraying. *Dig Endosc* 2024; **36**: 446-54.

3 Laine L, Kaltenbach T, Barkun A, McQuaid KR, Subramanian V, Soetikno R. Scenic international consensus statement on surveillance and management of dysplasia in inflammatory bowel disease. *Gastroenterology* 2015; **148**: 639-51.e28.

4 American Society for Gastrointestinal Endoscopy Standards of Practice C, Shergill AK, Lightdale JR *et al.* The role of endoscopy in inflammatory bowel disease. *Gastrointest Endosc* 2015; **81**: 1101-21 e1-13.

5 Lamb CA, Kennedy NA, Raine T *et al.* British society of gastroenterology consensus guidelines on the management of inflammatory bowel disease in adults. *Gut* 2019; **68**: s1-s106.

6 Murthy SK, Feuerstein JD, Nguyen GC, Velayos FS. Aga clinical practice update on endoscopic surveillance and management of colorectal dysplasia in inflammatory bowel diseases: Expert review. *Gastroenterology* 2021; **161**: 1043-51 e4.

7 Gordon H, Biancone L, Fiorino G *et al.* Ecco guidelines on inflammatory bowel disease and malignancies. *J Crohns Colitis* 2023; **17**: 827-54.

8 Kobayashi S, Fujimori T, Mitomi H *et al.* Immunohistochemical assessment of a unique basal pattern of p53 expression in ulcerative-colitis-associated neoplasia using computer-assisted cytometry. *Diagn Pathol* 2014; **9**: 99.

9 Matkowskyj KA, Chen ZE, Rao MS, Yang GY. Dysplastic lesions in inflammatory bowel disease: Molecular pathogenesis to morphology. *Arch Pathol Lab Med* 2013; **137**: 338-50.

10 Mutaguchi M, Naganuma M, Sugimoto S *et al.* Difference in the clinical characteristic and prognosis of colitis-associated cancer and sporadic neoplasia in ulcerative colitis patients. *Dig Liver Dis* 2019; **51**: 1257-64.

11 Geboes K, Riddell R, Ost A, Jensfelt B, Persson T, Löfberg R. A reproducible grading scale for histological assessment of inflammation in ulcerative colitis. *Gut* 2000; **47**: 404-9.

12 Bessissow T, Lemmens B, Ferrante M *et al.* Prognostic value of serologic and histologic markers on clinical relapse in ulcerative colitis patients with mucosal healing. *Am J Gastroenterol* 2012; **107**: 1684-92.
