# Supplementary material for: Adjustment of Surveillance Intervals for Ulcerative Colitis‐Associated Neoplasia Based on Disease Duration
Source: Dig Endosc. 2025 Jun 19;37(10):1068–77. doi: 10.1111/den.15073 (PMC12511916; doi:10.1111/den.15073)
Supplement: Supplementary file 2 — Table S1 Comparative characteristics of the 39 lesions included in the study, stratified by early‐stage neoplasia and advanced cancer. Table S2 Assumed lesion identification rates in each guideline based on disease duration. [file DEN-37-1068-s002.docx]

**Table S1. Comparative characteristics of the 39 lesions included in the study, stratified by early-stage neoplasia and advanced cancer.**

|  | Intramucosal or submucosal lesion (n = 25) | Advanced cancer (n = 14) | *P* value |
| --- | --- | --- | --- |
|  |  |  |  |
| **Male/Female**, n (%)/n (%) | 19 (76)/6 (24) | 8 (57)/6 (43) | 0.001 |
| **Age**, years, median (IQR) | 53(47–69) | 47(43–52) | 0.218 |
| **Disease duration**, years, median (IQR) |  |  | 0.616 |
| 8–14, n (%) | 7 (28) | 3 (21) |  |
| 15–19, n (%) | 7 (28) | 2 (14) |  |
| 20–24, n (%) | 2 (8) | 5 (36) |  |
| 25–29, n (%) | 5 (20) | 1 (7) |  |
| 30 or more, n (%) | 4 (16) | 3 (21) |  |
| **Surveillance interval**, years, median (IQR) | 1.4 (1.1–2.2) | 1.3 (1.0–1.5) | 0.633 |
| **Mayo endoscopic subscore** |  |  | 0.471 |
| 0, n (%) | 16 (64) | 4 (29) |  |
| 1, n (%) | 4 (16) | 8 (57) |  |
| 2, n (%) | 3 (12) | 2 (14) |  |
| 3, n (%) | 2 (8) | 0 (0) |  |
| **Concomitant primary sclerosing cholangitis**, n (%) | 0 (0) | 0 (0) |  |
| **Family history of colorectal cancer**, n (%) | 0 (0) | 0 (0) |  |
| **Stricture**, n (%) | 1 (4) | 1 (4) | 0.679 |
| **Pseudopolyposis**, n (%) | 6 (24) | 4 (29) | 0.761 |
| **Mucosal scarring**, n (%) | 14 (56) | 6 (43) | 0.444 |
| **Dysplasia <5 years**, n (%) | 4 (16) | 0 (0) | 0.120 |
| **Extent** |  |  | 0.484 |
| Pancolitis, n (%) | 19 (76) | 12 (86) |  |
| Left-sided colitis, n (%) | 6 (24) | 2 (14) |  |
| Proctitis, n (%) | 0 (0) | 0 (0) |  |
| **Morphology** |  |  | <0.001 |
| SCENIC classification |  |  |  |
| Pedunculated, n (%) | 1 (4) | 0 |  |
| Sessile, n (%) | 3 (12) | 0 |  |
| Superficial elevated, n (%) | 10 (40) | 0 |  |
| Flat, n (%) | 6 (24) | 0 |  |
| Depressed, n (%) | 5 (20) | 5 (36) |  |
| Macroscopic type |  |  |  |
| 1 (Polypoid type), n (%) | 0 (0) | 1 (7) |  |
| 2 (Ulcerative type with clear margin), n (%) | 0 (0) | 1 (7) |  |
| 3 (Ulcerative type with infiltration), n (%) | 0 (0) | 2 (14) |  |
| 4 (Diffusely infiltrating type), n (%) | 0 (0) | 0 (0) |  |
| 5 (Unclassified type), n (%) | 0 (0) | 5 (36) |  |
| **Tumor size**, mm, median (IQR) | 20 (15–31.5) | 60 (36–60) | <0.001 |
| **Location** |  |  | 0.348 |
| Ascending, n (%) | 1 (4) | 0 (0) |  |
| Transverse, n (%) | 0 (0) | 2 (14) |  |
| Descending, n (%) | 0 (0) | 2 (14) |  |
| Sigmoid, n (%) | 12 (48) | 3 (21) |  |
| Rectum, n (%) | 12 (48) | 7 (50) |  |
| **Histologic type** |  |  | <0.001 |
| High-grade dysplasia^†)^, n (%) | 18 (72) | 0 (0) |  |
| tub, n (%) | 5 (20) | 8 (57) |  |
| por/sig/muc, n (%) | 2 (8) | 6 (43) |  |

^†)^ Although neoplasms with nuclear and architectural abnormalities are diagnosed as intramucosal carcinoma regardless of invasion status in Japan, these lesions were classified as high-grade dysplasia in this study.

IQR, interquartile range

**Table S2. Assumed lesion identification rates in each guideline based on disease duration**

| Disease duration, years | Patient | Identification rate (Not adjusted), % | | | |
| --- | --- | --- | --- | --- | --- |
|  | n | ASGE | AGA | ECCO | BSG |
| 8–14 | 10 | 80 | 60 | 50 | 50 |
| 15–19 | 9 | 78 | 78 | 56 | 78 |
| 20–24 | 7 | 71 | 57 | 29 | 57 |
| 25–29 | 6 | 33 | 33 | 17 | 33 |
| 30 or more | 7 | 86 | 57 | 57 | 57 |
